# Supplementary material for: CircCDC42-encoded CDC42-165aa regulates macrophage pyroptosis in Klebsiella pneumoniae infection through Pyrin inflammasome activation
Source: Nat Commun. 2024 Jul 8;15:5730. doi: 10.1038/s41467-024-50154-x (PMC11231140; doi:10.1038/s41467-024-50154-x)
Supplement: Supplementary file 1 — Supplementary Information [file 41467_2024_50154_MOESM1_ESM.pdf]

Supplementary Materials for

CircCDC42 encoded CDC42-165aa regulates macrophage pyroptosis in *Klebsiella pneumoniae* infection through pyrin inflammasome activation

Authors: Nana Xu[1,2,#], Jiebang Jiang[1,#], Fei Jiang[1,3], Guokai Dong[1,2], Li Meng[1,2], Meng Wang[1,2], Jing Chen[1], Cong Li[4], Yongping Shi [1*], Sisi He[5,*], Rongpeng Li[1*]

Affiliations:

[1] Jiangsu Province Engineering Research Center of Cardiovascular Drugs Targeting Endothelial Cells, School of Life Sciences, Jiangsu Normal University, Xuzhou 221116, China;

[2] Laboratory of Morphology, Xuzhou Medical University, Xuzhou 221004, China;

[3] Department of Laboratory Medicine, the Affiliated Hospital of Xuzhou Medical University, Xuzhou 221002, China

[4] Xuzhou Key Laboratory of Emergency Medicine, the Affiliated Hospital of Xuzhou Medical University, Xuzhou 221002, China

[5] Department of Oncology, The Second Affiliated Hospital of Zunyi Medical University, Zunyi, China

[#]These authors contributed equally.

* Correspondence: lirongpeng@jsnu.edu.cn (R. Li), sisihe1219@163.com (S. He) or shiyongp@sina.com (Y. Shi)

This PDF file includes:

Supplementary Figure 1 to 7
Supplementary Table 1. Key Resources
Supplementary Table 2. The list of primer sequences used in the study

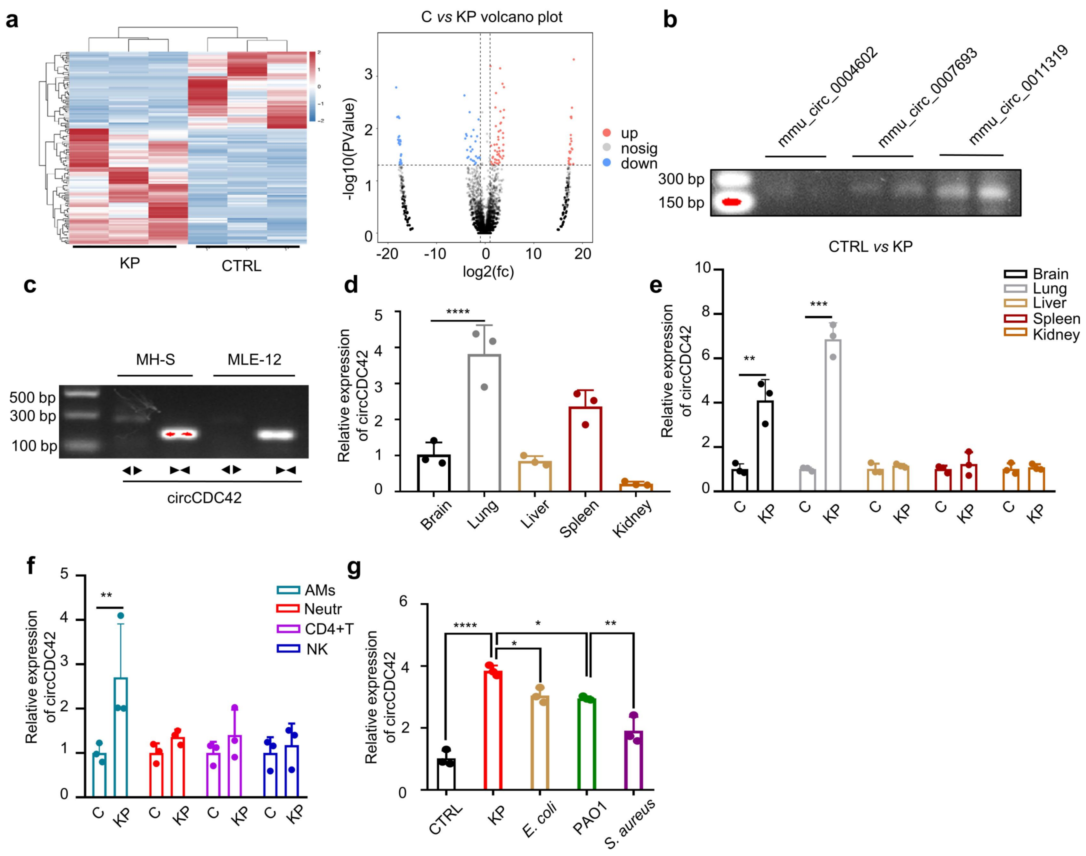

Supplementary Figure 1. CircRNAs screening in vivo and vitro after KP infection (Related to Figure 1)

a A total of 3 pairs of MH-S cell infected with or without KP (MOI of 20:1) were subjected to RNA sequencing (RNA-seq). Heatmap and volcano plot for microarray analysis of differentially expressed circRNAs in MH-S cells. **b** MH-S cells were infected with KP at an MOI of 20:1 for 4 h. cDNAs from MH-S were used as templates to amplify circRNAs using divergent primers. Related to Figure 1b (n = 3 biological replicates). **c** PCR products using circCDC42 divergent primers from MH-S and MLE-12 were analyzed by agarose gel electrophoresis (n = 3 biological replicates). **d** Expression levels of circCDC42 in the different tissues from C57BL/6J mice were examined by RT-qPCR (n = 3 biological replicates). **e** C57BL/6J mice were challenged intranasally with 5 × 10^6 colony forming units (CFU) of KP. Expression levels of circCDC42 in mice were examined by RT-qPCR (n = 3 biological replicates). **f** Expression levels of circCDC42 in the different cells were examined by RT-qPCR upon KP infection (n = 3 biological replicates). **g** Expression

levels of circCDC42 in mouse lungs were examined by RT-qPCR upon indicated pathogen (5×10^6 CFU) infection (n = 3 biological replicates). Data are shown as means ± SD. **d, g** One-way ANOVA with Tukey test. **e, f** Two-way ANOVA with Bonferroni's test. *$p < 0.05$, **$p < 0.01$, ***$p < 0.001$, ****$p < 0.0001$.

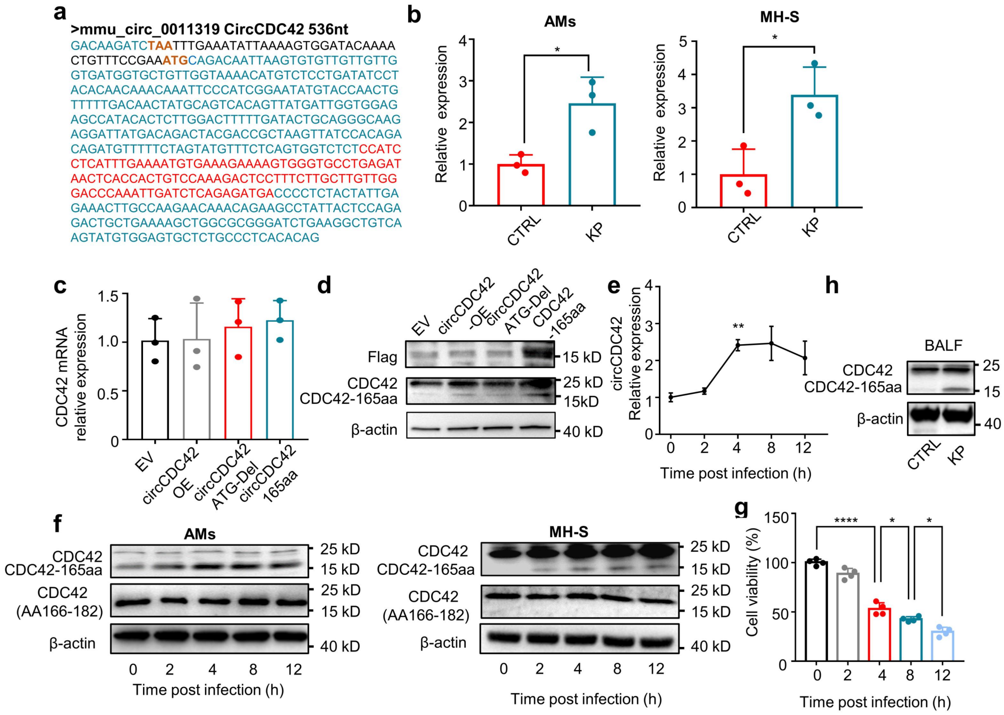

Supplementary Figure 2. Identification the unique CDC42-165aa sequence in circCDC42 (Related to Figure 2)

a The sequences of the putative ORF and IRES (red) are shown. Related to Figure 2a. **b** Primary AMs and MH-S cells were infected with KP at an MOI of 20 : 1 for 4 h. Relative expression of CDC42-165aa expression in AMs (left panel) and MH-S (right panel) as in Figure 2d (n = 3 biological replicates). **c** MH-S cells were treated with circCDC42 ATG delete plasmid, circCDC42 or CDC42-165aa plasmid. The linear CDC42 mRNA level was detected by RT-qPCR (n = 3 biological replicates). **d** Expression of CDC42-165aa-Flag and CDC42-165aa in the indicated MH-S were detected by immunoblotting (n = 3 biological replicates). **e** MH-S cells were infected with KP at an MOI of 20 : 1, and the level of circCDC42 was detected by RT-qPCR at the indicated time points (n = 3 biological replicates). **f** Primary AMs (left panel) and MH-S cells (right panel) were infected with KP at an MOI of 20 : 1. Expression of CDC42 and CDC42-165aa proteins in cells for indicated time point were detected by immunoblotting using CDC42 antibody (Affinity, DF6322, AA51-74) or CDC42 antibody (Santa Cruz, SC-8401, AA166-182) (n = 3 biological replicates). **g** MH-S cells were infected with KP at an MOI of 20 : 1 for indicated time point, and cell viability was measured by MTT assay (n = 4 biological replicates). **h**

C57BL/6J mice were challenged intranasally with 5×10^6 CFU of KP for 24 h. Expression levels of CDC42-165aa in AMs isolated from BALF of mice with KP infection for 24 h were examined by immunoblotting (n = 3 biological replicates). Data are shown as means ± SD. **b** Unpaired two-tailed t-test. **c, e, g** One-way ANOVA with Tukey test. $*p < 0.05$, $**p < 0.01$, $***p < 0.001$, $****p < 0.0001$.

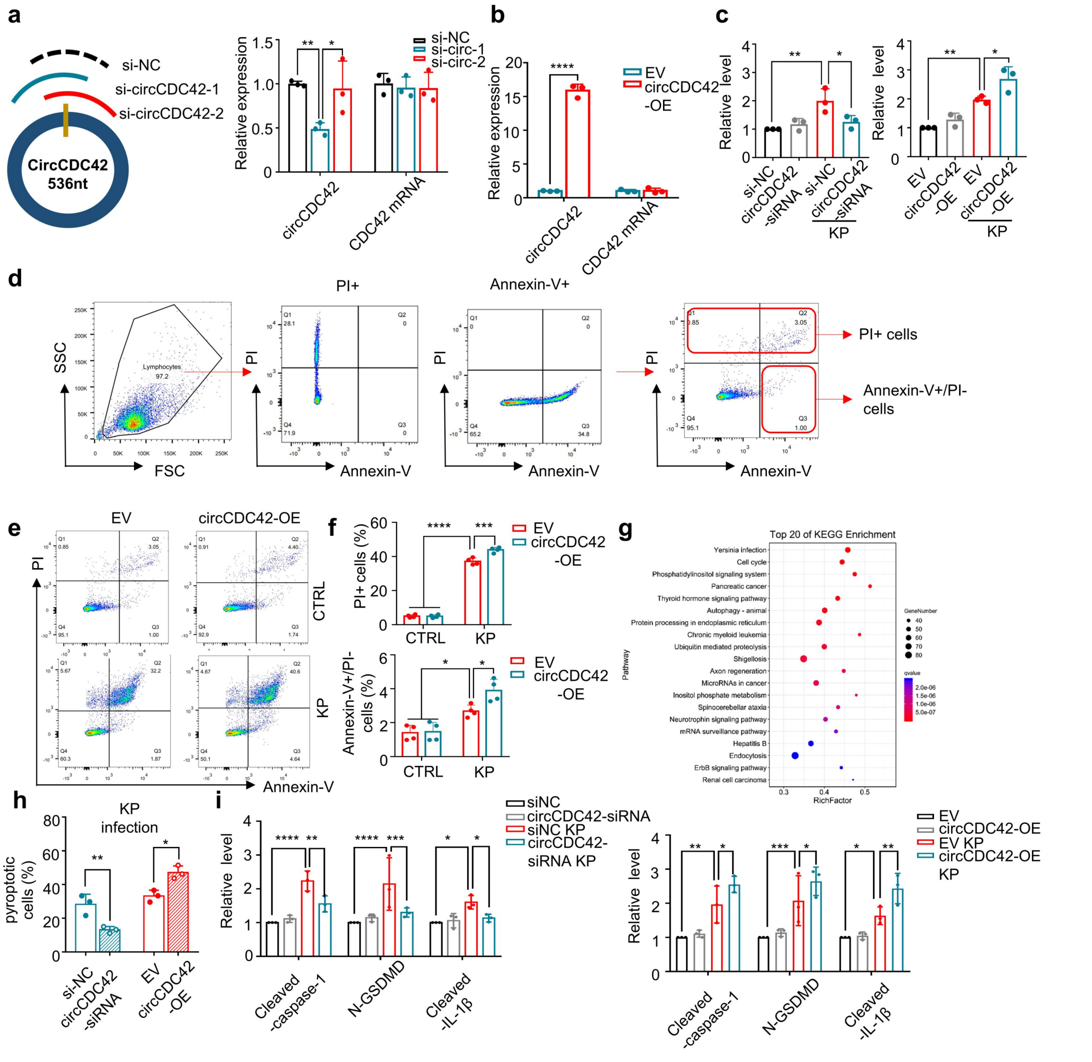

Supplementary Figure 3. Related to Figure 3.

a Left, illustration of the circCDC42 junction-specific FISH probe and circCDC42 siRNA target site. Right, MH-S cells were transfected with siNC or circCDC42 siRNA, circCDC42 and linear CDC42 mRNA level were detected by RT-qPCR (n = 3 biological replicates). **b** MH-S cells were transfected with empty vector (EV) or circCDC42 overexpreesion (OE) plasmid, respectively for 24 h. circCDC42 and linear CDC42 mRNA level were detected by RT-qPCR (n = 3 biological replicates). **c** The indicated MH-S cell swere infected with KP at an MOI of 20:1 for 4 h. Relative amounts of CDC42-165aa expression in MH-S cells treated as

in Figure 3a (n = 3 biological replicates). **d** Annexin-FITC and PI staining of MH-S cells. The gating strategy for the MH-S cells (Annexin V+PI^- cells and PI^+ cells). **e** Annexin-FITC and PI staining of MH-S cells subjected to KP infection with or without overexpreesion (OE) plasmid treatment. Representative FACS plots are shown (n = 3 biological replicates). **f** A summary of the percentage of Annexin V+PI^- cells and PI^+ cells among dead cells as in Supplementary Figure 3d (n = 3 biological replicates). **g** Enrichment of differentially expressed genes in different pathways by KEGG analysis. **h** MH-S cells were transfected with EV or circCDC42-OE plasmids, respectively for 24 h, then infected with KP at an MOI of 20:1 for 4 h. The percentage of pyroptotic cells in Figure 3d was calculated (n = 3 biological replicates). **i** MH-S cells were transfected with siNC or circCDC42 siRNA, EV or circCDC42-OE plasmid, respectively for 24 h. The cells were then infected with KP at an MOI of 20:1 for 4 h. Relative level of Cleaved-Caspase-1, N-GSDMD and Cleaved-IL-1β in MH-S cells treated as indicated in Figure 3f (n = 3 biological replicates). Data are shown as means ± SD. **a, b, c, f, h, i** Two-way ANOVA with Bonferroni's test. *$p < 0.05$; **$p < 0.01$; ***$p < 0.001$; ****$p < 0.0001$.

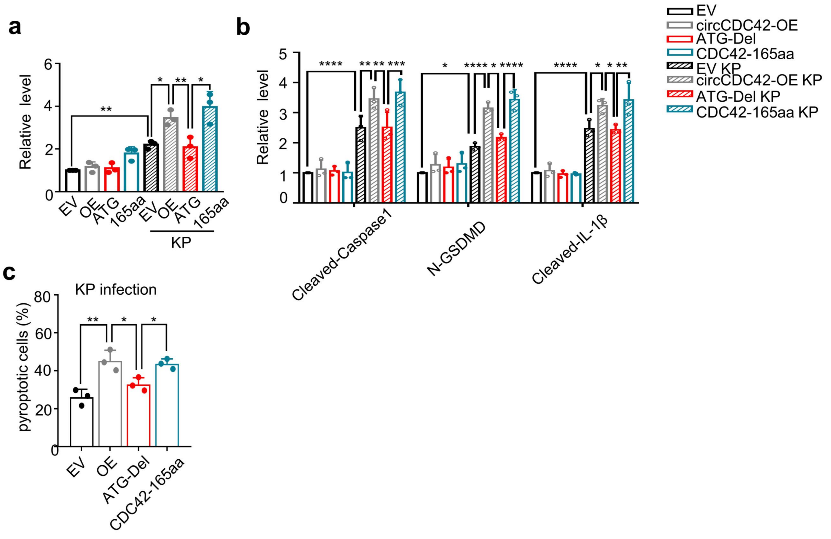

Supplementary Figure 4. Related to Figure 4 MH-S cells were transfected with EV, circCDC42-OE plasmid, circCDC42 ATG-Del plasmid, or CDC42-165aa plasmid, respectively for 24 h. **a** Relative level of CDC42-165aa expression in MH-S cells treated as indicated in Figure 4a (n = 3 biological replicates). **b** The percentage of pyroptotic cells in Figure 4c was calculated (n = 3 biological replicates). **c** Relative level of cleaved-Caspase-1, N-GSDMD and cleaved-IL-1β in MH-S cells treated as indicated in Figure 4e (n = 3 biological replicates). Data are shown as means ± SD. **a, c** Two-way ANOVA with Bonferroni's test. **b** One-way ANOVA with Tukey test. *$p < 0.05$; **$p < 0.01$; ***$p < 0.001$; ****$p < 0.0001$.

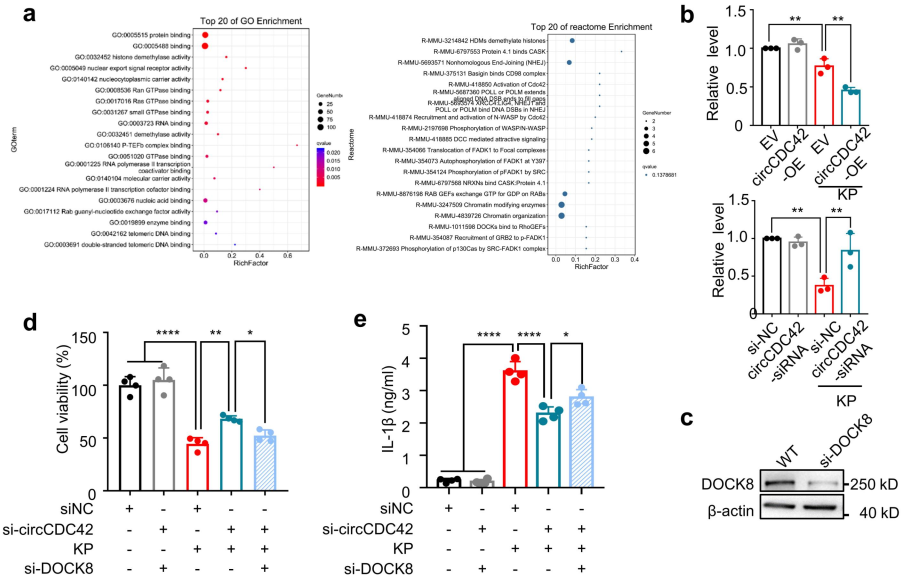

Supplementary Figure 5. Related to Figure 5

a A total of 3 pairs of MH-S cell samples infected with or without KP (MOI of 20 : 1) were subjected to RNA sequencing (RNA-seq). Enrichment of differentially expressed genes by Reactome and GO analysis. **b** MH-S cells were transfected with empty EV, circCDC42-OE plasmid, circCDC42 ATG-Del plasmid, or CDC42-165aa plasmid, respectively for 24 h. Cells were then infected with KP at an MOI of 20 : 1 for 4 h. Relative level of activated GTPases CDC42 in MH-S cells treated as indicated in Figure 5e (n = 3 biological replicates). **c** MH-S cells were treated with or without DOCK8 siRNA for 24 h. DOCK8 levels in MH-S cells were detected by western blotting (n = 3 biological replicates). **d** The indicated MH-S cells were infected with or without KP (MOI of 20 : 1) for 4 h, and cell viability was measured by MTT assay (n = 4 biological replicates). **e** The secretion of IL-1β in cell supernatant after KP infection (MOI of 20 : 1) for 4 h was detected by ELISA (n = 4 biological replicates). Data are shown as means ± SD. **b, d, e** One-way ANOVA with Tukey test. *$p < 0.05$; **$p < 0.01$; ***$p < 0.001$; ****$p < 0.0001$.

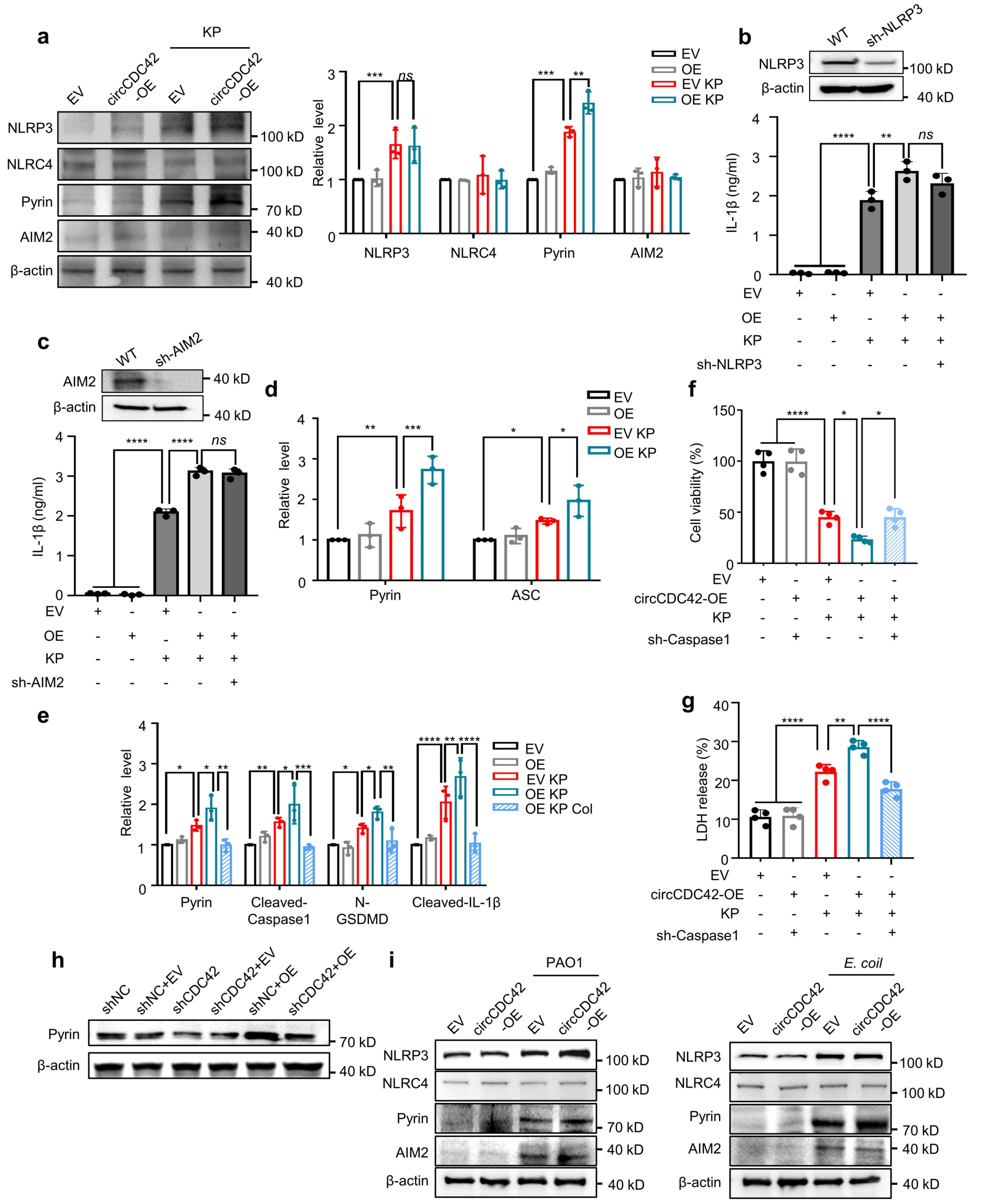

Supplementary Figure 6. Related to Figure 6

a MH-S cells were transfected with EV or circCDC42-OE plasmid, respectively for 24 h, then infected with KP at an MOI of 20 : 1 for 4 h. Left, expression of NLRP3, NLRC4, Pyrin, and AIM2 in MH-S cells were detected by immunoblotting. Right, relative levels were quantified by ImageJ (n = 3 biological replicates). **b** MH-S cells were treated with EV, circCDC42 -OE plasmid, or NLRP3 shRNA as indicated for 24

h, then infected with KP (MOI of 20 : 1, 4 h) or not. Western Blotting showing NLRP3 expression in MH-S cells. The secretion of IL-1β in cell supernatant was detected by ELISA (n = 3 biological replicates). **c** MH-S cells were treated with EV, circCDC42 -OE plasmid, or AIM2 shRNA as indicated for 24 h, then infected with KP (MOI of 20:1, 4 h) or not. AIM2 level in MH-S cells was detected by western blotting. The secretion of IL-1β in cell supernatant was detected by ELISA (n = 3 biological replicates). **d** The expression level of Pyrin bound to ASC was assessed using optical density and presented as fold change to EV group, as shown in Figure 6a (n = 3 biological replicates). **e** Relative level of Pyrin, cleaved-Caspase-1, N-GSDMD, and cleaved-IL-1β in MH-S cells treated as indicated, shown as in Figure 6e (n = 3 biological replicates). **f** MH-S cells were treated with EV, circCDC42 -OE plasmid, or Caspase-1 shRNA for 24 h, then infected with or without KP (MOI of 20 : 1) for 4 h. The cell viability was measured by MTT assay (n = 5 biological replicates). **g** LDH release was measured from MH-S cells infected with or without KP (MOI of 20:1) for 4 h , and presented as percent of medium activity compared to cell lysate from cells (n = 4 biological replicates). **h** MH-S cells were treated with EV, circCDC42 -OE plasmid, or CDC42 shRNA as indicated for 24 h, then infected with KP (MOI of 20:1) for 4 h. Pyrin level in MH-S cells was detected by western blotting. (n = 3 biological replicates). **i** Protein expression of NLRP3, NLRC4, Pyin, and AIM2 in MH-S cells transfected with indicated plasmid with PAO1 or *E. coli* infection (MOI of 20 : 1) for 4 h or not (n = 3 biological replicates). Data are shown as means ± SD. **a, d, e** Two-way ANOVA with Bonferroni's test. **b, f, g** One-way ANOVA with Tukey test. **p*

< 0.05; **$p < 0.01$; ***$p < 0.001$; ****$p < 0.0001$, *ns* no significant difference.

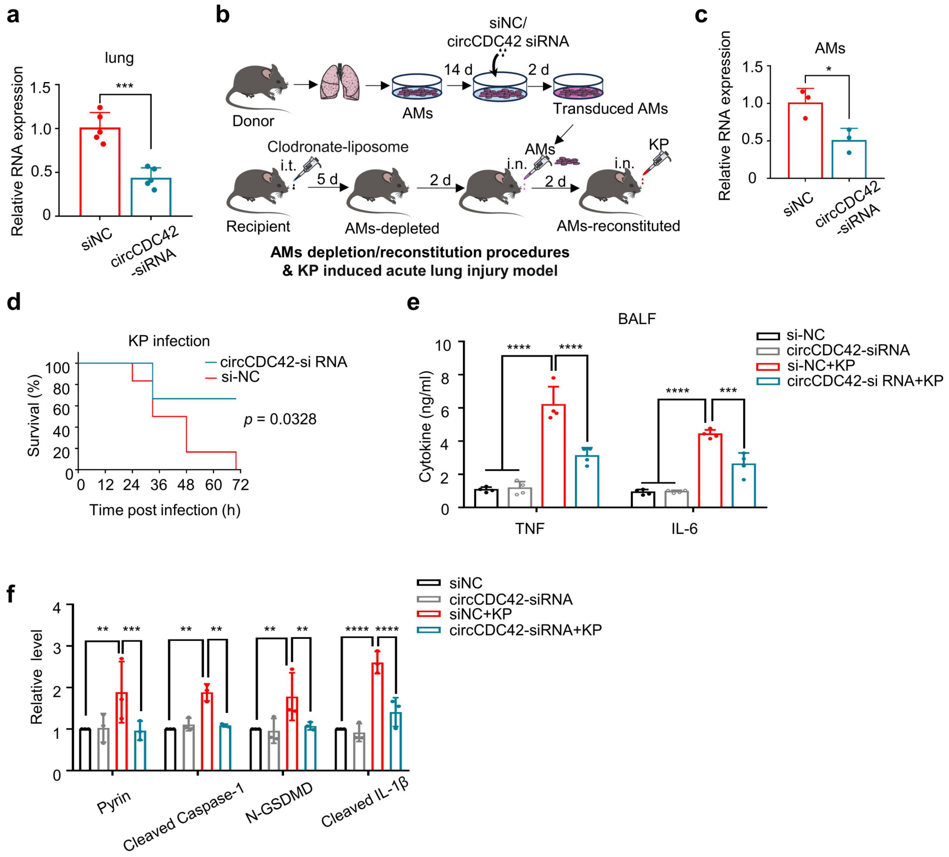

Supplementary Figure 7. Related to Figure 7

a Moues model of KP infection was established as Figure 7a shown. RT-qPCR analysis of circCDC42 expression in lungs from KP-infected siNC- and circCDC42-siRNA- injected mice (n = 5). **b** AMs depletion/reconstitution procedures and KP induced acute lung injury model. **c** RT-qPCR analysis of circCDC42 expression in AMs from BLAF in Figure S7b mice (n = 3 biological replicates). **d** Kaplan-Meier survival curves of KP-infected siNC- and circCDC42 siRNA-AMs Recipient mice (n = 6). Survival was determined up to 72 h (p = 0.0328). **e** Expression levels of TNF and IL-6 in BLAF from KP-infected siNC- and circCDC42 siRNA-AMs Recipient mice were determined by ELISA (n = 4 biological replicates). **f** Relative level of Pyrin, cleaved-Caspase-1, N-GSDMD, and cleaved-IL-1β in AMs

from KP-infected siNC- and circCDC42 siRNA-AMs Recipient mice, as shown in Figure 7h (n = 3 biological replicates). Data are shown as means ± SD. **a, c** Unpaired two-tailed t-teste. Log-rank (Mantel-Cox) test. **e, f** Two-way ANOVA with Bonferroni's test. **p* < 0.05; ***p* < 0.01; ****p* < 0.001; *****p* < 0.0001.

Supplementary Table 1. Key Resources

REAGENT or RESOURCE	SOURCE	IDENTIFIER
**Antibodies**		
Caspase-1 (E2Z1C) Rabbit mAb (1:1000)	Cell Signaling Technology	#24232
Cleaved Gasdermin D (Gly277) Antibody (1:1000)	Cell Signaling Technology	#34667
NLRP3 (D4D8T) Rabbit mAb (1:1000)	Cell Signaling Technology	#15101;
IL-1β (3A6) Mouse mAb (1:1000)	abcam	#ab234437
ASC/TMS1/PYCARD Antibody (B-3) (1:1000)	Santa Cruz	#sc-514414
Anti-Pyrin antibody (1:1000)	abcam	#ab195975
AIM2 Polyclonal antibody (1:1000)	Proteintech	#20590-1-AP
DOCK8 Polyclonal antibody (1:1000)	Proteintech	#11622-1-AP
Beta Actin Monoclonal antibod (1:5000)	Proteintech	#66009-1-Ig
Cdc42 Antibody (B-8)（166-182aa）(1:1000)	Santa Cruz	#sc-8401
m-IgG3 BP-HRP (1:1000)	Santa Cruz	#sc-533670
CDC42 Rabbit Polyclonal Antibody (1:1000)	Affinity Biosciences	#DF6322
ANTI-FLAG mouse Monoclonal antibody mouse (1:1000)	Sigma-Aldrich	#F1804-50UG
NLRC4 Rabbit pAb (A7382) (1:1000)	ABclonal	#A7382
CoraLite488-conjugated Goat Anti-Mouse IgG(H+L) (IF 1:100)	Proteintech	#SA00013-1
CoraLite594-conjugated Goat Anti-Rabbit IgG(H+L) (IF 1:100)	Proteintech	#SA00013-4
HRP-conjugated Affinipure Goat Anti-Mouse IgG(H+L) (WB 1:5000)	Proteintech	#SA00001-1
HRP-conjugated Affinipure Goat Anti-Rabbit IgG(H+L) (WB 1:5000)	Proteintech	#SA00001-2
CD170 (Siglec F) mAb (IF 1:100)	Thermo Fisher Scientific	#14-1702-82
**Critical Commercial Assays**		
Mouse TNF-alpha ELISA Kit	Proteintech	#KE10002
Mouse IL-6 ELISA Kit	Proteintech	#KE10007
Enhanced BCA Protein Assay Kit	Beyotime	#P0010
Lacate Dehydrogenase(LDH) Activity Assay Kit	Solarbio	#BC0685
Ultra-High Sensitivity ECL Kit	MCE	#HY-K1005
Cdc42 Pull-down Activation Assay	Cytoskeleton	# BK034
The Dual-Luciferase Reporter Assay System	Promega	#E1910

Supplementary Table 2. The list of primer sequences used in the study

Name	Sequence (5' to 3')
For quantitative real-time PCR	
circCDC42-F (divergent)	ATGTGGAGTGCTCTGCCCTC
circCDC42-R(divergent)	TCCTCTTGCCCTGCAGTATCAA
circCDC42-F (convergent)	GGATTATGACAGACTACGACCGCTAAG
circCDC42 -R(convergent)	GGCAGAGCACTCCACATACTTGAC
circRanbp9-F	ACCCGGCAAAAGCAGCACTG
circRanbp9-R	AAGCGGTCTATCTGGGCCTG
circSgms1-F	TGGGCGTTTTCTATTTGCGA
circSgms1-R	ACACTATCCCTCCTTGGCTG
IL-6-F	TTCTTGGGACTGATGCTGGT
IL-6-R	CTGTGAAGTCTCCTCTCCGG
TNF-F	TCAGGCGATCTTCCCATCTC
TNF-R	AAGTGGGATGGTTGGTAGGG
IL-1β-F	CGTTCCCATTAGACAGCTGC
IL-1β-R	TCAGCTCATATGGGTCCGAC
GAPDH-F (divergent)	GCTGAGTATGTCGTGGAGTCT
GAPDH-R (divergent)	GGCAGCCCTGGTGACCAGGCGC
GAPDH-F (convergent)	GAGCGAGACCCCACTAACAT
GAPDH-R (convergent)	CCCTTCCACAATGCCAAAGTT
U6-F	TGCTCGCTTCGGCAGCACAT
U6-R	CTTGCGCAGGGGCCATGCTA
Oligonucleotides for FISH	
circCDC42 FISH	Cy3-AGATCTTGTCCTGTGTGAGGGCAG-Cy3
For SiRNA/SHRNA sequences	
circCDC42-siRNA-1	AUCUUGUCCUGUGUGAGGGCATT
circCDC42-siRNA-2	AAUUAGAUCUUGUCCUGUGUGTT
si NC	UUCUCCGAACGUGUCACGUTT
si-Dock8	GGCTTGTACGAGACGGTTAAT
sh-AIM2	TCCCAGGATTAGTAAACTGAA
sh-Caspase1	CCATGTTGGATCAGATCAACT
sh-NLRC4	CAGAAATTGAAGCCCTGATAA
sh-NC	CCCGAACGGCACGTT
