# Supplementary material for: CircCDC42-encoded CDC42-165aa regulates macrophage pyroptosis in Klebsiella pneumoniae infection through Pyrin inflammasome activation
Source: Nat Commun. 2024 Jul 8;15:5730. doi: 10.1038/s41467-024-50154-x (PMC11231140; doi:10.1038/s41467-024-50154-x)
Supplement: Supplementary file 3 — Reporting Summary [file 41467_2024_50154_MOESM3_ESM.pdf]

nature portfolio

Corresponding author(s): Rongpeng Li (NCOMMS-23-40201B)

Last updated by author(s): May 13, 2024

Reporting Summary

Nature Portfolio wishes to improve the reproducibility of the work that we publish. This form provides structure for consistency and transparency in reporting. For further information on Nature Portfolio policies, see our Editorial Policies and the Editorial Policy Checklist.

Statistics

For all statistical analyses, confirm that the following items are present in the figure legend, table legend, main text, or Methods section.

n/a	Confirmed	
☐	☒	The exact sample size (*n*) for each experimental group/condition, given as a discrete number and unit of measurement
☐	☒	A statement on whether measurements were taken from distinct samples or whether the same sample was measured repeatedly
☐	☒	The statistical test(s) used AND whether they are one- or two-sided<br>*Only common tests should be described solely by name; describe more complex techniques in the Methods section.*
☒	☐	A description of all covariates tested
☒	☐	A description of any assumptions or corrections, such as tests of normality and adjustment for multiple comparisons
☐	☒	A full description of the statistical parameters including central tendency (e.g. means) or other basic estimates (e.g. regression coefficient) AND variation (e.g. standard deviation) or associated estimates of uncertainty (e.g. confidence intervals)
☐	☒	For null hypothesis testing, the test statistic (e.g. *F*, *t*, *r*) with confidence intervals, effect sizes, degrees of freedom and *P* value noted<br>*Give P values as exact values whenever suitable.*
☒	☐	For Bayesian analysis, information on the choice of priors and Markov chain Monte Carlo settings
☒	☐	For hierarchical and complex designs, identification of the appropriate level for tests and full reporting of outcomes
☒	☐	Estimates of effect sizes (e.g. Cohen's *d*, Pearson's *r*), indicating how they were calculated

Our web collection on statistics for biologists contains articles on many of the points above.

Software and code

Policy information about availability of computer code

Data collection	qRT-PCR data were collected and analyzed by the ABI 7500 Fast Dx instruments Sequence Detection Software v2.0.4; Western blot images were captured using Bio-Rad Image Lab software v 5.2.1; Microscopy images were captured by Olympus IX71 microscope; Immunofluorescence and FISH, fluorescent images were acquired using Leica STELLARIS 5 confocal microscopy;
Data analysis	Microsoft excel, Graph Pad Prism 7.0, Image J software V1.8.0.112, ORFfiner (https://www.ncbi.nlm.nih.gov/orffinder), IRESfinder (https://github.com/xiaofengsong/IRESfinder)

For manuscripts utilizing custom algorithms or software that are central to the research but not yet described in published literature, software must be made available to editors and reviewers. We strongly encourage code deposition in a community repository (e.g. GitHub). See the Nature Portfolio guidelines for submitting code & software for further information.

Data

Policy information about availability of data

All manuscripts must include a data availability statement. This statement should provide the following information, where applicable:

- Accession codes, unique identifiers, or web links for publicly available datasets
- A description of any restrictions on data availability
- For clinical datasets or third party data, please ensure that the statement adheres to our policy

CircCDC42 was recorded in circBase database (http://www.circbase.org/).The RNA-seq analysis datasets generated during this study are available through the NCBI

Sequence Read Archive under the Bioproject PRJNA984001 identifier. The protein mass spectrometry raw data have been deposited to the ProteomeXchange Consortium via the iProX partner repository with the dataset identifier PXD045396. The remaining data are available within the Article and Supplementary Information.

Research involving human participants, their data, or biological material

Policy information about studies with human participants or human data. See also policy information about sex, gender (identity/presentation), and sexual orientation and race, ethnicity and racism.

Reporting on sex and gender	This study did not involve human participants.
Reporting on race, ethnicity, or other socially relevant groupings	This study did not involve human participants.
Population characteristics	This study did not involve human participants.
Recruitment	This study did not involve human participants.
Ethics oversight	This study did not involve human participants.

Note that full information on the approval of the study protocol must also be provided in the manuscript.

Field-specific reporting

Please select the one below that is the best fit for your research. If you are not sure, read the appropriate sections before making your selection.

☒ Life sciences ☐ Behavioural & social sciences ☐ Ecological, evolutionary & environmental sciences

For a reference copy of the document with all sections, see nature.com/documents/nr-reporting-summary-flat.pdf

Life sciences study design

All studies must disclose on these points even when the disclosure is negative.

Sample size	No sample size calculation wer performed. The sample sizes were determined based on previous literature (doi.org/10.1038/nmicrobiol.2016.132),as well as allowing for statistical analyses such as calculation of standard deviation and performing t-tests. For animal studies, sample size was chosen to comply with the 3R principles to minimize the number of mice used. In vitro studies were repeated a minimum of three times for independence.
Data exclusions	No data were excluded from the analyses in this study
Replication	Except for some annimal studies stated otherwise in legends, each experiments were applied with at least 3 biological replications with similiar results.All details on biological and technical replicates are provided in the text or figure legends.
Randomization	Allocation was random.
Blinding	For in vivo studies, investigators were not blinded to the animal experiments as ensuring a success lung infection models required professional knowledge and experience. For sequencing data processing and quality control, the investigator in charge was blinded to sample condition.

Behavioural & social sciences study design

All studies must disclose on these points even when the disclosure is negative.

Study description	
Research sample	
Sampling strategy	
Data collection	
Timing	
Data exclusions	

Non-participation

Randomization

Ecological, evolutionary & environmental sciences study design

All studies must disclose on these points even when the disclosure is negative.

Study description

Research sample

Sampling strategy

Data collection

Timing and spatial scale

Data exclusions

Reproducibility

Randomization

Blinding

Did the study involve field work? ☐ Yes ☐ No

Field work, collection and transport

Field conditions

Location

Access & import/export

Disturbance

Reporting for specific materials, systems and methods

We require information from authors about some types of materials, experimental systems and methods used in many studies. Here, indicate whether each material, system or method listed is relevant to your study. If you are not sure if a list item applies to your research, read the appropriate section before selecting a response.

Materials & experimental systems

n/a	Involved in the study	
☐	☒	Antibodies
☐	☒	Eukaryotic cell lines
☒	☐	Palaeontology and archaeology
☐	☒	Animals and other organisms
☒	☐	Clinical data
☒	☐	Dual use research of concern
☒	☐	Plants

Methods

n/a	Involved in the study	
☒	☐	ChIP-seq
☐	☒	Flow cytometry
☒	☐	MRI-based neuroimaging

Antibodies

Antibodies used

anti-caspase1(WB,1:1000),CST,#24232
anti-cleaved GASDMD (WB, 1:1000),CST,#34667
anti-NLRP3 (WB,1:1000),CST,#15101
anti-IL-1b (WB,1:1000),abcam,#ab234437

anti-ASC (WB,1:1000),Santa Cruz,#sc-514414
anti-AIM2 (WB,1:1000),Proteintech #20590-1-AP
anti-Pyrin (WB, 1:1000),abcam,#ab195975
anti-DOCK8 (WB,1:1000), Proteintech,#11611-1-AP
anti-beta actin (WB, 1:5000), Proteintech,#66009-1-AP
anti-cdc42(wb,1:1000),santa cruz,#sc-8401
m-IgG3 BP-HRP (1:1000),santa cruz,#sc-533670
anti-cdc42 (wb,1:1000),affinity,#DF6322
anti-flag (wb,1:2000), sigma,#F1804
anti-NLRC4 (wb, 1:1000),ABclonal,#A7382
anti-mouse IgG (wb,1:5000),Proteintech,#SA00001-1
anti-rabbit IgG (wb,1:5000),Proteintech,#SA00001-2
coralite488-conjugated anti mouse IgG (IF,1:500),Proteintech,#SA00013-1
coralite594-conjugated anti rabbit IgG (IF,1:500),Proteintech,#SA00013-4
anti-CD170(Siglec F) (IF,1:100),Thermo,#14-1702-82
Information of antibodies used in this study were also provided in supplementary Table 1.

Validation

All commercially available antibodies were validated by vendors. Validation statements are provided on the manufacture's website. We examined primary antibodies according to manuals, and got similar results with validation results on manufacturer's website or relevant citations.

Eukaryotic cell lines

Policy information about cell lines and Sex and Gender in Research

Cell line source(s)

Murine alveolar macrophages cell line (MH-S) and murine type II lung epithelial cell line (MLE-12) were acquired from ATCC. Murine Pimary CD4+T cell, neutrophil (MNHC) and NK cell used in this study were described in Methods section.

Authentication

Cell lines were obtained from original sources and were not further authenticated.

Mycoplasma contamination

Cell lines used in the study were tested negative for mycoplasma contamination.

Commonly misidentified lines
(See ICLAC register)

No commonly misidentified cell lines were used in the study.

Palaeontology and Archaeology

Specimen provenance

Specimen deposition

Dating methods

☐ Tick this box to confirm that the raw and calibrated dates are available in the paper or in Supplementary Information.

Ethics oversight

Note that full information on the approval of the study protocol must also be provided in the manuscript.

Animals and other research organisms

Policy information about studies involving animals; ARRIVE guidelines recommended for reporting animal research, and Sex and Gender in Research

Laboratory animals

C57BL/6J mice(age:7-8 weeks), weighing 18-22 g, were purchased from GemPharmatech Co., Ltd. (Nanjing, China). Mice were maintained on a 12-h light/dark cycle in a temperature-controlled environment (22-25 °C) and free access to food and water at the Center of Experimental Animals of Xuzhou Medical University.

Wild animals

No wild animals were used in the study.

Reporting on sex

Only male mice were used in the study. Since female mice must be tested across the estrous cycle and are more variable than males, male mice were used in this proof-of-concept study.

Field-collected samples

No field-collected samples were used in the study.

Ethics oversight

All protocols used in this study were approved by the Institutional Animal Care and Treatment Committee of the Xuzhou Medical University, Approval no. 202306T017.

Note that full information on the approval of the study protocol must also be provided in the manuscript.

Clinical data

Policy information about clinical studies

All manuscripts should comply with the ICMJE guidelines for publication of clinical research and a completed CONSORT checklist must be included with all submissions.

Clinical trial registration

Study protocol

Data collection

Outcomes

Dual use research of concern

Policy information about dual use research of concern

Hazards

Could the accidental, deliberate or reckless misuse of agents or technologies generated in the work, or the application of information presented in the manuscript, pose a threat to:

No	Yes	
☐	☐	Public health
☐	☐	National security
☐	☐	Crops and/or livestock
☐	☐	Ecosystems
☐	☐	Any other significant area

Experiments of concern

Does the work involve any of these experiments of concern:

No	Yes	
☐	☐	Demonstrate how to render a vaccine ineffective
☐	☐	Confer resistance to therapeutically useful antibiotics or antiviral agents
☐	☐	Enhance the virulence of a pathogen or render a nonpathogen virulent
☐	☐	Increase transmissibility of a pathogen
☐	☐	Alter the host range of a pathogen
☐	☐	Enable evasion of diagnostic/detection modalities
☐	☐	Enable the weaponization of a biological agent or toxin
☐	☐	Any other potentially harmful combination of experiments and agents

Plants

Seed stocks

Novel plant genotypes

Authentication

ChIP-seq

Data deposition

☐ Confirm that both raw and final processed data have been deposited in a public database such as GEO.

☐ Confirm that you have deposited or provided access to graph files (e.g. BED files) for the called peaks.

Data access links
May remain private before publication.

Files in database submission

Genome browser session (e.g. UCSC)

Methodology

Replicates

Sequencing depth

Antibodies

Peak calling parameters

Data quality

Software

Flow Cytometry

Plots

Confirm that:

☒ The axis labels state the marker and fluorochrome used (e.g. CD4-FITC).

☒ The axis scales are clearly visible. Include numbers along axes only for bottom left plot of group (a 'group' is an analysis of identical markers).

☒ All plots are contour plots with outliers or pseudocolor plots.

☒ A numerical value for number of cells or percentage (with statistics) is provided.

Methodology

Sample preparation	Cells were filtered twice through 70 um filter, then stained with fluorophore-conjugated antibodies for 15 minutes at room temperature. The excess of unbound antibodies was washed out before acquisition in flow cytometry
Instrument	BD FASCSanto ?
Software	Flow cytometry data was analyzed with FlowJo V10.
Cell population abundance	Purity of the sorted cells was not assessed post-sorting due to the limited numbers of sorted cells. In addition, the markers assessed would not be expressed by any other cell types potentially present.
Gating strategy	No-stained negative controls were used in the experiments to define gating.

☒ Tick this box to confirm that a figure exemplifying the gating strategy is provided in the Supplementary Information.

Magnetic resonance imaging

Experimental design

Design type

Design specifications

Behavioral performance measures

Acquisition

Imaging type(s)

Field strength

Sequence & imaging parameters

Area of acquisition

Diffusion MRI ☐ Used ☐ Not used

Preprocessing

Preprocessing software	
Normalization	
Normalization template	
Noise and artifact removal	
Volume censoring	

Statistical modeling & inference

Model type and settings	
Effect(s) tested	

Specify type of analysis: ☐ Whole brain ☐ ROI-based ☐ Both

Statistic type for inference (See Eklund et al. 2016)	
Correction	

Models & analysis

n/a	Involved in the study
☐	☐ Functional and/or effective connectivity
☐	☐ Graph analysis
☐	☐ Multivariate modeling or predictive analysis

Functional and/or effective connectivity	
Graph analysis	
Multivariate modeling and predictive analysis	
